# Disclosing Pharmacogenetic Feedback of Caffeine via eHealth Channels, Assessment of the Methods and Effects to Behavior Change: A Pilot Study

**DOI:** 10.3389/fdgth.2020.567656

**Published:** 2021-02-09

**Authors:** Kerti Alev, Andres Kütt, Margus Viigimaa

**Affiliations:** ^1^Digital Health, Tallinn University of Technology (TalTech), Tallinn, Estonia; ^2^Information Technology and Communication Technologies, Information Technology Department, Tallinn University of Technology (TalTech), Tallinn, Estonia; ^3^North Estonia Medical Center, Tallinn University of Technology, Tallinn, Estonia

**Keywords:** mHealth, pharmacogenetics, behavior change, Caffeine, eHealth, decision support, digital health, mobile

## Abstract

**Background:** The integration of genetic testing into eHealth applications holds great promise for the personalization of disease prevention guidelines. However, relatively little is known about the impact of eHealth applications on an individual's behavior.

**Aim:** The aim of the pilot study was to investigate the effect of the personalized eHealth application approach to behavior change in a 1-month follow-up period on groups with previously known and unknown caffeine impacts.

**Method:** We created a direct-to-consumer approach that includes providing relevant information and personalized reminders and goals on the digital device regarding the caffeine intake for two groups of individuals: the intervention group (IG) with the genetic raw data available and the control group (CG) to test the impact of the same content (article about caffeine metabolism) on participants without the genetic test. Study participants were all Estonians (*n* = 160).

**Results:** The study suggests that eHealth applications work for short-term behavior change. Participants in the genetic IG tended to increase caffeine intake if they were informed about caffeine not being harmful. They reported feeling better physically and/or mentally after their behavioral change decision during the period of the study.

**Conclusions:** Our pilot study revealed that eHealth applications may have a positive effect for short-term behavior change, regardless of a prior genetic test. Further studies among larger study groups are required to achieve a better understanding about behavior change of individuals in the field of personalized medicine and eHealth interventions.

## Introduction

With the development of interactive devices and extensive connectivity to the web, the online channel has become popular to deliver tailored healthy lifestyle-promoting interventions. In general, online behavioral health change programs (eHealth channels) seem to work very well-according to those studies and meta-analyses (1–5). eHealth channels act as an important factor of self-efficacy and patient education, and are essential to the improvement of patient–clinician communication, along with improvement in trust, adherence, and social support (6).

The Mayo Clinic observed understanding and perspectives on pharmacogenomics for patients in 2017: how their patients understood the effect of their personalized medicine. It was discovered that 30% of the participants did not understand the results, and further work is needed to establish a better understanding of the results (7). It was found that a chunking strategy could help genetic feedback participants better obtain information that they did not relate to before, even better if the participants obtained it in an extended period (8). Also, performance feedback and self-monitoring techniques have given good results in behavior change programs (9). Focused goal setting, when it is combined with personal feedback messages, is also considered a promising approach (9).

Many studies noted that more frequent communication could improve behavioral health outcomes. Minimal contact will not help to boost smoking cessation (10). Orleans et al. suggested that more frequent follow-up phone calls increased the cessation by up to 50%. According to Hietaranta-Luoma et al. the positive effect of lifestyle change seemed to fade during the “silent” period of the trial. According to Brouwer et al., the most used behavior-changing techniques were feedback, interactive elements, and email/phone contact (add reference). Furthermore, the most effective techniques were listed as peer support, counselor support, email/phone contact, and updates of the intervention website. Schmidlen et al. suggest that genetic counselors offer health topics for the participants to choose from based on their interest and offer visual aids to explain the risk scores. Another systematic review revealed that without lifestyle counseling, the reaction to behavioral change can be modest (11). After online genetic counseling in the breast cancer study, the willingness to adapt to a healthier lifestyle after genetic testing results was high among women who participated in the study for breast cancer risk (12). A study trial plan has been made to define the steps in behavioral change after the direct-to-consumer genetic testing (DTCGT). The results could lead to a better understanding of behavior change and DTCGT in the future (13).

We studied individuals with and without previous genetic test and offered them a tool or method for behavior change in a commonly used medicine and nutrition ingredient—caffeine. Caffeine belongs to our everyday products, but some tend to drink it more than others. Caffeine consumption is partially genetically regulated by metabolism of the gene CYP1A2 [we used the single-nucleotide polymorphism (SNP) rs762551].

While the study participants were organized into a group with their genetic information [intervention group (IG) and a group without genetic test [control group (CG)], both groups were offered the same information about caffeine: how it is related to genetics, metabolism, and caffeine health risks. Both groups were offered digital tools (mobile app MediKeep^1^ with a notification system and plain email-based reminders), set goals, and reminders for behavior change (online web form to choose preferences).

This leads to the topic of the current study. This study assesses if genetic test makes DTCGT participants change their behavior in the short term (1-month follow-up) when using eHealth services with strategic methods: information chunking, creating personalized reminders and goals on their digital device.

This study has the following sub-aims:

1. Whether people with genetic test results (when reading the article about caffeine genetic health traits and risks) tend to decide to change their behavior compared to the people reading the same article without a genetic test.
2. Does the genetic test result help people to decide to increase specific nutrition if they find it genetically suitable for them?
3. Does using goals and reminders help people using eHealth service to change their behavior in the short term (1-month follow-up)?

## Materials and Methods

This pilot study consists of the assessment of the channels and methods based on the previous studies (**Table 2**) and an empirical study among *n* = 160 participants in Estonia. The authors conducted a study, based on the previous findings (**Table 2**), to test the hypothesis among DTCGT participants (IG) and the same content impact on participants (CG) without any prior knowledge of their genetic metabolism for caffeine.

The study has been approved by the Research Ethics Committee of the University of Tartu (approval no.: 290/T-10 since 8.03.2019 until 30.06.2019).

### Methods and Channels for eHealth and Genetic Feedback

With the development of interactive devices and extensive connectivity to the web, the online channel has become a popular way to deliver tailored healthy lifestyle-promoting interventions. In general, online behavior health change programs seem to work very well according to studies and meta-analyses (1–5). The importance of self-efficacy and patient education is essential to improve patient–clinician communication, along with improvement in trust, adherence, and social support (6).

However, a study among Food4Me participants found no effect in physical activity change between groups with genetic test and fat mass- and obesity-associated (FTO) genetic risk. The personalized feedback led to improved self-reported physical activity outcomes in general, suggesting that personalized advice rules out genetic risk score (1).

According to Brouwer et al. (4), the most used behavior-changing techniques were feedback, interactive elements, and email/phone contact. Furthermore, the most effective techniques were listed as peer support, counselor support, email/phone contact, and updates of the intervention website.

In the REVEAL trials, the study group followed the people for a whole year after learning their genomic risks (14). What they learned, in general, was that giving out graphics or illustrating the risk scores on the timelines along with the genetic counseling in their practice was the best way of doing it. They were not claiming it to be the absolute best methodology. Artificial intelligence (AI) and smartphone-based motivation and action support system can improve and maintain the physical activity among adult populations while they got actively engaged and reminded about the program (15).

However, in a personalized medicine setting, problems were found in understanding the results. The Mayo Clinic observed understanding and perspectives on pharmacogenomics for patients in 2017: how their patients understood the effect of their personalized medicine. It was discovered from the results that one third did not understand the results, and further work is needed to establish a better understanding of results (7). A chunking strategy could help genetic feedback participants better obtain the information that they did not relate to before, even better if they obtained it over a more extended period (8).

Performance feedback and self-monitoring techniques have given good results in behavior change programs (9). Focused goal setting, when combined with personal feedback messages, is also considered a promising approach (9).

Many studies noted that more frequent communication could improve behavior health outcomes. Minimal contact will not help boost smoking cessation (10). Orleans et al. (16) suggested that more frequent follow-up phone calls increased the cessation by up to 50%. According to Hietaranta-Luoma et al. (17), the positive effect of lifestyle change seemed to fade during the “silent” period of the trial.

Schmidlen et al. (18) suggest that genetic counselors offer health topics for the participants to choose from based on their interest and offer visual aids to explain the risk scores. Another systematic review revealed that without lifestyle counseling, the reaction to behavior change can be modest (11). After online genetic counseling in the breast cancer study, the willingness to adapt to a healthier lifestyle after genetic testing results was high among women who participated in the study for breast cancer risk (12). A study trial plan has been made to define the steps in behavior change after the DTCGT. The results could lead to a better understanding of behavior change and DTCGT in the future (13).

### Behavioral Change and eHealth

While there are guides for health services or tools for best practices like Cochrane reviews and NICE guidance, the behavior change factors were more effectively described in “The behavior change wheel: A new method for characterizing and designing behavior change interventions” by Michie et al. (19). Those functions could be easily translated as methods to the eHealth application format.

According to this “behavior change wheel,” the change starts with three components: capability, opportunity, and motivation—the COMB-B system (20).

- **Intervention** functions surround the base components. To improve change, the deficit among intervention functions should be decreased as suggested by the article.
- **Education** stands for increasing the knowledge or understanding.
- **Persuasion** means using communication.
- **Incentivization** is creating an expectation of a reward.
- **Coercion** indicates punishment.
- Other functions are listed as training, restriction, environmental restructuring, and enablement.

Edwards et al. (21) found that among mobile applications which were using gamification in mobile health, the most popular techniques were self-regulatory. Those included several proven health behavior change methods like goal setting, self-monitoring, and feedback (21).

### Literature Overview: Assessment of the Channels and Methods Based on Previous Studies

In November 2018, the words “DNA test” and “behavior change” were searched on the Google.com search platform in Estonia by the authors of this study. An article came into interest, “Genetic testing does not change how most people behave, study finds”^2^, leading to the original systematic review and meta-analysis in BMJ.com, published in 2016 (22). Rather than searching for individual studies for the current research, the BMJ meta-analysis was used to assess if other factors are contributing to DNA test and behavior change.

The trials were eligible for the BMJ 2016;352:i1102 study if they were randomized controlled trials or quasi-randomized controlled trials among adults and included one group that got personalized DNA risk estimates for risks where the behavior could change the health risk outcome. The analysis includes 18 clinical trials around the world: the United States (8), the United Kingdom (5), Japan (3), Finland (1), and Canada (1). Behaviors included smoking, alcohol consumption, diet, and physical activity.

Table 1 lists all included studies (*n* = 19), with close to 6,000 participants in total, while Table 2 is a checklist and shows what kind of channels and methods were used in the study. The authors were not able to fill all the blocks because of missing or unclear information in the study, and such cells were marked with a question mark (?).

**Table 1:** BMJ 2016;352:i1102 included clinical trials, their number, topic, and number of participants.

**No.**	**Name/Year**	**Topic**	**No. of participants**
1	Audrain et al. (10)	Smoking	426
2	Hishida et al. (23)	Smoking	562
3	Ito et al. (24)	Smoking	617
4	McBride et al. (25)	Smoking	557
5	Sanderson et al. (26)	Smoking	61
6	Chao et al. (14)	Medicine or supplements	162
7	Hendershot et al. (27)	Reduced alcohol	200
8	Hietaranta-Luoma et al. (17)	Reduced alcohol	107
9	Komiya et al. (28)	Reduced alcohol	329
10	Glanz et al. (29)	Sun protection	73
11	Chao et al. (14)	Diet	162
12	Godino et al. (30)	Diet (Diabetes II)	557
13	Hietaranta-Luoma et al. (17)	Diet	107
14	Marteau et al. (31)	Diet	341
15	Meisel et al. (32)	Diet	279
16	Nielsen et al. (33)	Diet	138
17	Voils et al. (34)	Diet	601
18	Weinberg et al. (35)	Screening and behavior programs	783
19	Grant et al. (36)	Screening and behavior programs	177

*[Note that (17) and (14) are represented in two categories]*.

**Table 2:** BMJ 2016;352:i1102 included clinical trials in meta-analysis, their interactions, and their communication channels.

	**Study no./Topics**	**1**	**2**	**3**	**4**	**5**	**6**	**7**	**8**	**9**	**10**	**11**	**12**	**13**	**14**	**15**	**16**	**17**	**18**	**19**
	Positive change in intervention group				x		x			x		x		x			x			
	Positive change in any group	x	-	x	?	x	x	x	x	x	x	x	?	x	-	-	x	-	?	x
Interactions	Three or more follow-ups	-	-	-	-	-	-	-	x	-	-	-	-	x	-	-	-	-	-	-
	Reminders	-	-	-	x	x	-	-	-	-	-	?	-	-	-	-	x	-	-	x
	Rewards	-	-	-	x	-	-	-	-	-	x	-	-	-	-	-	?	x	-	x
	Increased knowledge	x	-	x	x	x	x	x	x	-	x	x	-	x	?	x	x	x	x	x
	Printed materials	-	x	-	x	x	x	-	-	-	x	x	x	-	-	x	-	x	-	x
	Increased motivation	x	-	-	x	x	-	x	x	x	-	?	?	x	-	?	?	-	-	x
Channels	Face to face	x	-	x	-	x	x	-	x	x	x	?	-	x	x	-	-	x	x	x
	Email	-	-	x	-	-	-	x	x	-	-	?	-	x	-		x	-	x	-
	Telephone	x	-	-	x	x	-	-	-	-	x	?	-	-	x	-	-	-	x	-
	Webpage/online interaction	-	-	-	-	-	-	x	-	-	-	?	-	-	-	-	x	-	x	-

The major finding from Table 2 indicates that while the BMJ 2016;352:i1102 study focused on the change between CG and IG and mostly no change was found, it did reveal that there was a positive change in most of the groups. It confirmed the assumption that while people's education and knowledge were raised about the topic, positive behavioral change was also found after participating in the trial/program.

It was also suggested that some topics might be more motivational and easier to act on for the participants to take behavioral actions. For example, while willingness to increase physical activity was higher among the FTO gene risk group, actual results in physical activity did not rise (32). However, taking supplements for Alzheimer's prevention is considered an easier task than regular gym visits or changes in diet (14).

Only Hietaranta-Luoma et al. (17) had three or more follow-ups. Clear “reminder” techniques were detected in four of the studies (25, 26, 33, 36), while it was uncertain in one (14). Physical or beneficial rewards were present in four of the studies (25, 29, 34, 36), while it was not available in one (33). Most of the trials increased knowledge of the participants, while it was not done in two (23, 30), resulting in no effect on health behavior in any of those groups. More than half of the trials offered printed materials, 11 offered face-to-face counseling or sessions with doctors, seven had communications via email at least once, six trials included a phone call, and only three had some kind of online interaction or communication. Increased motivation for behavioral change was detected in seven of the trials.

The studies had several limitations, from small study groups to not well-targeted participants. For example, college students (mean age 22) might not be interested in reducing body fat or they might not drink alcohol as much to reduce their drinking in general (32).

### Empirical Study Design

#### Subjects

The subjects were all native Estonians, and all spoke the Estonian language. They were all adults (≥18 years) recruited online via social media and forum advertisements with targeted keywords: caffeine, genetic testing, digital technology, and personalized medicine. The previous genetic test was not a prerequisite to participate in the study. If the participant had a previous DTCGT raw data available, they were asked to provide the information.

#### Study Design

Invitation to the study was sent out via social media channels, local forums, and Facebook groups in Estonia (#Tervis, #uhkegeenidoonor, and #MediKeep). The questionnaires were published on the Tyeform.com platform, which allows fluid usability and logic jumps in online questionnaire forms. The questionnaires and study design graphics are provided as Supplementary Material for this article.

The questionnaires were conducted based on Nielsen et al.'s (37) initial research questions for “A randomized trial of genetic information for personalized nutrition,” The questionnaire was slightly modified for the empirical study, including the option to set a goal and reminder (9) besides the third questionnaire for self-assessment of the behavior health results after 1-month follow-up.

Participants were organized into groups based on their first questionnaire. Participants with previous genetic testing results and in whom CYP1A2 gene SNP rs762551 was available were organized into an IG “with genetic test results,” while those “without a genetic test” conducted comprised the CG. They were also organized by the preference of the next communication channel: mobile app MediKeep, email, or both. If the subjects were not sure about having their DNA raw data, additional email was sent to clarify the answer. If the answer confirmed the genotype results, the participant was added to the IG. If the answer was negative and they did not know their caffeine metabolism from any of the testing companies, the participant was assigned to the CG. For file transfer, additional service was provided via the MASV^3^ portal for fast file delivery solution, especially when handling large files. For security reasons, raw data files were signed with id.ee^4^ services (encryption to be sent only for the author's national ID or digital signature).

After the successful first questionnaire, the groups received an email or mobile app message to read an article about genetics and caffeine metabolism in the Estonian language. The IG received their results with information about their genotype, while the CG was offered to read the article without their genetic profile.

The second questionnaire was linked just below the article. The second online form asked the participants if they understood the article, if it had any new information for them in regard to caffeine, and how they related to the results. Participants were asked if they would like to change anything in their health behaviors and if they would like to set a goal and reminders. Again, two channels were offered: by email or via a MediKeep mobile application push-notification system.

If no goals or reminders were set, participants were directed to the final question informing about the third questionnaire to be sent in the 1-month follow-up. Participants who opted-in for goals and reminders were also asked if they would like to increase or decrease their caffeine intake and if they had any specific favorites (coffee, green tea, cacao, chocolate, or anything else). They were informed that the reminders would be sent no more than five times a week for 1 month. The reminders were set manually for every participant based on their answer to get a more personalized meaning, while also trying to mimic AI. They were set to be sent out automatically in a 3-day interval but no more than 10 times per participant for 1 month.

The reminders were also set by time, in the morning, or at lunch, depending on personal preferences. The message texts were changed slightly once a week to prevent reminder exhaustion and spam, while the goal remained the same. Participants were reminded at least three times by email if they had not answered their second questionnaire in 3 weeks. The data management was done in Typeform.com^5^ and imported to Google Spreadsheet in March–May 2019, and all duplicate responses were deleted after new response information was confirmed.

The third questionnaire was sent out to every participant who had finished the second questionnaire with or without the goals/reminders set. The last questionnaire asked if they had decided to change something in their behavior and when. Questions also included decision making, whether genetic test results had any impact in deciding what to change, physical and mental well-being, whether reminders and goals had any impact (with or without genetic test), and whether they would have liked to get similar topics about other genetic traits. The questionnaire also had one open-ended question for additional comments.

The results were collected in the Typeform.com platform and data collected in Google Spreadsheet. The calculations were made using the Chi-Square Calculator for a 2 × 2 contingency table^6^; take note that every cell should have a number of 1 and 20% of the cells should not have data <5 (38).

## Results

### Questionnaires

Compared to the first questionnaire, the actual completion rate was 80.75% for the second one (Table 3). The third questionnaire received a completion rate of 93% compared to the second, and from all the participants who started the questionnaire, 75% completed the third and final questionnaire.

**Table 3:** Questionnaire participation rates and devices in Typeform.com.

	**Responses**	**Total visits**	**Unique visits**	**Completion rate%**	**Average time to complete**
First questionnaire	213	1,364	1,111	19.3	10:33
PCs and laptops	121	689	549	22.20	10:40
Smartphones	83	635	526	15.80	10:51
Tablets	9	40	36	25	06:15
Second questionnaire	172	359	314	55.1	03:14
PCs and laptops	107	182	143	75.5	02:56
Smartphones	62	169	154	40.3	03:52
Tablets	3	8	7	24.9	03:06
Completion rate compared to the first questionnaire				80.75	
Third questionnaire	160	372	180	88.9	10:44
PCs and laptops	85	181	91	93.4	17:54
Smartphones	68	176	82	82.9	02:39
Tablets	7	15	7	100	02:32
Completion rate compared to the second questionnaire				93	
Completion rate compared to the first questionnaire				75	

The first questionnaire received 1,111 unique visits, resulting in 213 responses by the 1st of May 2019 (one duplication removed from 214 total responses). The completion rate was 19.3%, and it took an average of 10:33 min to finish the questionnaire.

All participants were native Estonian speakers. Majority of the participants were female 79.9%, *n* = 167 and aged between 30 and 39 (39.9%, *n* = 85), and only one belonged to the 70–79 age group (Table 4). Males were 20.1%, *n* = 42. There was a demographic difference in the IG where males were more represented (*p*-value = 0.0012).

**Table 4:** Sample demographics according to the first questionnaire.

**Age groups**	**Intervention group**	**Control group**	***p***
18–29	8	38	0.43
30–39	23	62	0.11
40–49	10	48	0.34
50–59	4	14	0.94
60–69	1	4	
70 and more	0	1	
Education			
Primary	1	5	
Secondary	12	53	0.46
Bachelor's degree	18	73	0.57
Master's degree	13	35	0.22
Doctoral degree	2	1	
Gender			
Female, *n* = 167	29	138	0.0012
Male, *n* = 42	17	25	

Most of the respondents had at least a bachelor degree (42.7%, *n* = 91). Only 19.7% of the participants had not heard anything about DTCGT company tests before, 57.3% (*n* = 122) had heard somewhat, and 23% (*n* = 49) had heard a lot. Only 5% (*n* = 10) had heard a lot about the term nutrigenomics, and 25.8% (*n* = 55) had not discovered the term or field of science previously.

Majority of the participants (63.5%, *n* = 134) were genuinely interested in the relations between diet and genetics, while one participant was definitely not (0.5%). Even more of them agreed that there is a benefit in learning about how genetic makeup affects diet (73.8%, *n* = 157), 22.5% (*n* = 48) somewhat agreed, 2.8% (*n* = 6) neither agreed nor disagreed, and 0.9% (*n* = 2) would rather not disagree.

Most of the participants (43.2%, *n* = 92) were somewhat assured that learning about genetic makeup will affect what they eat, 38% (*n* = 81) agreed very much, 16.4% (*n* = 35) neither agreed nor disagreed, and 2.3% (*n* = 5) disagreed.

Participants somewhat disagreed (54.7%, *n* = 116) that the results would make them uncomfortable and anxious to learn about the genetic findings, 22.2% (*n* = 47) neither agreed nor disagreed, 20.8% (*n* = 44) somewhat agreed, and 2.4% (*n* = 5) were sure they would.

Most of them (82.5%, *n* = 175) were sure a genetic test would make them learn more about themselves, 9.4% (*n* = 20) somewhat agreed, 5.2% (*n* = 119) neither agreed nor disagreed, and 2.8% (*n* = 6) would rather not agree. However, a little bit less were ready to change anything in their behavior to be healthier (66.7%, *n* = 142), 23% (*n* = 49) somewhat agreed, 7% (*n* = 15) neither agreed or disagreed, and 3.3% (*n* = 7) would rather disagree.

They agreed (66.7%, *n* = 140), somewhat agreed (23%, *n* = 49), neither agreed nor disagreed (7%, *n* = 15), and disagreed (4.2%, *n* = 9) to take the genetic test for the doctor to monitor their health more closely.

Participants preferred the next communication channel to be via email (80.6%, *n* = 170), while 13.3% (*n* = 28) preferred both email and mobile and 6.2% (*n* = 13) preferred mobile only.

Over half of the respondents (58.7%, *n* = 125) did not have their DNA raw data, 20.7% (*n* = 44) were not sure they had the data, and 20.7% (*n* = 44) confirmed they had the data. The high percentage of participants who were “not sure” was related to the next question asking for the testing company name, where a high percentage who answered the “Other” option were Estonian Biobank participants, and they were not sure if they had their results or DNA raw data. Those people were organized into the CG instead, and other obscure answers were corrected, with additional information requested *via* email. After correction, 21.5% (*n* = 46) in total had confirmed to have their DNA data, and everyone else was organized into the “no DTCGT group” 78.5% (*n* = 167). After correction of the testing company name, *n* = 38 of the people with raw genomic data were previous clients of MediKeep OÜ, and about *n* = 8 were willing to share their data from other companies (23andme.com, FTDNA, and Geni.com).

Those participants decided to send their files through email or just looked up the marker information and copied the required rows from the DNA raw data file as plain text via email. No one from the eight participants (outside from the MediKeep client base with their raw data) used the Massive.io portal or signed/encrypted their data as described in the study introduction and suggested in the email. An additional comment from one of the emails was “I hope this file comes through. I do not worry much about my data security—so there you are!”

It was impossible to assess whether those who did not respond to the email asking about their DNA data received the email in the first place, as this email was sent from a private email address with no tracking capabilities. All MediKeep clients had their information already within the company and agreed with the study consent to look at their genotypes—resulting in a high percentage of participation in the study after the second questionnaire until the third (100%).

The second questionnaire received 172 results (314 unique visits) with a completion rate of 55.1%, and it took an average of 03:14 min to finish, not including the time to read the article and for decision making (Table 3). Compared to the first questionnaire, the second one had a completion rate of 80.75%. It was impossible to measure how much time the participants took for decision making. However, the majority completed the questionnaire on the same day when they received it.

Generally, the participants (75%, *n* = 129) did not feel uneasy after reading the article, 16.9% (*n* = 29; *n* = 2 with genetic test results) did feel uneasy, and 8.1% (*n* = 14) did not know how to answer. Almost all (98.3%, *n* = 169) understood what the caffeine article was explaining, while 1.7% (*n* = 3) did not fully understand the content; they also did not have a previous genetic test. Over half of the respondents (69.2%, *n* = 119) found the article information to be something new, while 30.8% (*n* = 53) did not find it new to their knowledge. From *n* = 172 participants who completed the second survey, 36% (*n* = 62) opted in for goals and reminders, and 64% (*n* = 110) did not. From the CG, *n* = 48 opted in, and from the IG, *n* = 14 opted in (Table 5).

**Table 5:** Sub-aim 1: differentiation not found between the IG and CG regarding the decision to change current caffeine consumption with the help of set goals and reminders (*p* = 0.80).

	**Intervention**	**Control**	**Marginal row totals**
Reminder	14 (13.7) [0.01]	48 (48.3) [0]	62
No reminder	24 (24.3) [0]	86 (85.7) [0]	110
Marginal column totals	38	134	172 (grand total)

*Table information is provided as follows: observed cell totals (expected cell totals) [chi-square statistic for each cell]*.

From the reminder group, 72.6% (*n* = 44) preferred the reminder channel to be email, and 27.4% (*n* = 18) chose the mobile application for the channel (*n* = 8 Android and *n* = 10 iOS operating platforms). Android users did not receive their messages within the first week because of technical problems, and their participation in setting reminders was postponed for 1 week.

While 71% (*n* = 44) wished to decrease their coffee consumption, nobody wished to decrease or increase cacao consumption (Table 6). However, the other option included reduction of cola products. The products to be increased were coffee (*n* = 4), (green) tea (*n* = 3), and chocolate (*n* = 1). For the one participant who wished to increase chocolate consumption, the authors of this study sent a personalized message indicating that the participant may increase chocolate intake as they wished; however, it was suggested to choose at least 70% cocoa/dark chocolate for better health behavior.

**Table 6:** Sub-aim 2: participants with DTCGT may increase the caffeine intake, based on the knowledge about their caffeine metabolism (the *p* = 0.047. This result is significant at p < 0.05); however, the sample size is quite limited.

	**Intervention**	**Control**	**Marginal Row Totals**
Increase caffeine	4 (1.81) [2.66]	4 (6.19) [0.78]	8
Decrease caffeine	10 (12.19) [0.39]	44 (41.81) [0.12]	54
Marginal column totals	14	48	62 (grand total)

*The table information is provided as follows: observed cell totals (expected cell totals) [chi-square statistic for each cell]*.

The third questionnaire received *n* = 160 results. From 180 unique visitors, 88.9% completed the questionnaire compared to the second and third questionnaires, which had completion rates of 93 and 75%, respectively (Table 3). The questionnaire received *n* = 38 answers in IG and *n* = 122 in CG. Twenty-four participants in IG received their caffeine test results five or more months ago (63% from the 38 of the total in IG). Four participants did not receive their reminder messages due to technical reasons.

In general, 40 participants out of 160 answered how they felt after 1-month follow-up: one felt better physically, 10% (*n* = 4) felt better mentally, 25% (*n* = 10) felt better physically and mentally, 25% (*n* = 10) did not feel better and 37.5% (*n* = 15) did not know how to answer.

For the control question asking if they had set the goals and reminders, 59.1% (*n* = 95) answered no, 27.7% (*n* = 44) confirmed they did, and 13.2% (*n* = 21) did not recall whether they did or not.

Sixty-five people answered how long they had followed their reminder messages on a 1–5 scale. Over a quarter (38.5%, *n* = 25) “did not follow at all,” equivalent to “1” on the scale. Seven (10.8%) answered “2,” while nine (13.8%) answered “3.” Reminders were well followed by 15.4% (*n* = 10), equivalent to “4” on the scale; and 21.5% (*n* = 14) answered “5.”

Twenty-two people (33.8%) did not open the reminder messages at all (mobile or email), while 24.6% (*n* = 16) opened all messages. The middle groups scoring “2–4” were, respectively 15.4%, *n* = 10; 13.8%, *n* = 9; and 12.3%, *n* = 8.

Half of the people (50%, *n* = 66) thought reminders have been or would have been helpful to support behavior change, while 35.2% (*n* = 56) did not know how to answer and 23.9% (*n* = 38) thought they would not have been helpful.

Whether genetic test would have helped toward behavior change was supported by 71.8% (*n* = 115) in general, 22.6% (*n* = 36) did not know whether it would have helped, and 5.7% thought it would not have helped. People were also positively minded to receive other genetic trait information on a similar basis, where 80.6% (*n* = 129) agreed, 13.8% (*n* = 22) did not know how to answer, and 5.7% (*n* = 9) disagreed.

Some demographic differences were detected (Table 7). The age group 30–39 was generally more represented (*p* = 0.02) compared to the other age groups in the third questionnaire. In the IG, there were statistical differences, where male participants were more represented (*p* = 0.033).

**Table 7:** Sample demographics according to the third questionnaire.

	**Intervention**	**Control**	***p***
Female	23 (28.02) [0.9]	95 (89.98) [0.28]	0.033
Male	15 (9.98) [2.53]	27 (32.02) [0.79]	
**Age**			
18–29	8 (9.38) [0.2]	31 (29.62) [0.06]	0.55
30–39	21 (14.91) [2.49]	41 (47.09) [0.79]	0.02
40–49	6 (9.38) [1.22]	33 (29.62) [0.39]	0.14
50–59	3 (3.37) [0.04]	11 (10.63) [0.01]	0.8
60–69	0	3	
70–79	0	1	
**Education**			
Primary	1	2	
Secondary	11 (12.19) [0.12]	40 (38.81) [0.04]	0.63
Bachelor's degree	16 (16.01) [0]	51 (50.99) [0]	0.99
Master's degree	8 (8.36) [0.02]	27 (26.64) [0]	0.86
Doctoral degree	2	1	

*Table information is provided as follows: observed cell totals (expected cell totals) [chi-square statistic for each cell]*.

Setting of personalized goals and reminders was managed by special software, integrated into the MediKeep mobile application; additionally, it had the functionality to send automated emails. In some cases, the emails sent via this service ended up in junk email, and according to the third questionnaire and the “Technical question: did the reminders reach your email box or smartphone messages,” two participants from both IG and CG did not receive the “reminder” messages (they were also removed from the statistical analysis). All participants were receiving the reminders within 1 month and at least 10 times and 3 days apart. Reminder exhaustion was reduced by manually changing the message text but not the context. Some participants received their messages in the morning while some did in the afternoon, depending on their personal preferences (example reminder at 1 PM: “Do not take another coffee cup in the afternoon and dinner! You will get better sleep at night!”).

### Statistical Calculations

The aim of the study was to assess if genetic tests made people change their behavior in the short term (1-month follow-up) when using eHealth services with strategic methods: information chunking and creating personalized reminders and goals. To calculate the results, a chi-square test is performed.

Participants who decided not to change their behavior or did not see the need to change were removed from both groups. Additionally, two participants who wished to change their behavior were removed from both groups for technical reasons, as they did not receive their reminders. The total of participants in the final calculations were *n* = 18 (47.3% of 38) in the IG and *n* = 49 (40.1% of 122) in the CG.

#### Testing the Aim of the Study

We did not detect differences between the IG group with set reminders changing their behavior in the short term (*p* = 0.19) and the other groups (Table 8). There were no differences in gender for those groups (*p* = 0.32). According to the qualitative data—a test of association—no cell should have data <1, and 20% of the cells should have data of 5 (38).

**Table 8:** Test of the study hypothesis that genetic tests make DTCGT participants change their behavior in the short term (1-month follow-up) when using eHealth services with strategic methods: information chunking and creating personalized reminders and goals on their digital device.

	**IG (with reminders)**	**CG (including IG without reminders)**	**Marginal row totals**
Changed behavior	8 (6.03) [0.64]	26 (27.97) [0.14]	34
Did not change	3 (4.97) [0.78]	25 (23.03) [0.17]	28
Marginal column totals	11	51	62 (grand total)

*There is no association found with the p = 0.19. Table information is provided as follows: observed cell totals (expected cell totals) [chi-square statistic for each cell]*.

#### Other Findings

No differences were found between the groups who decided to change their behavior related to caffeine in the first place and those who did not (based on the second questionnaire, the *p*-value is 0.91). Participants in the IG felt better mentally or physically or both (*p*-value = 0.024) after adjusting their caffeine behavior after 1-month follow-up (Table 9).

**Table 9:** Incidental finding: participants in the intervention group felt better (mentally or physically) after adjusting their caffeine intake with set goals and reminders after 1 month (*p* = 0.024).

	**IG**	**CG**	**Marginal row totals**
Felt better	8 (4.8) [2.13]	8 (11.2) [0.91]	16
Did not feel better or did not know how to answer	4 (7.2) [1.42]	20 (16.8) [0.61]	24
Marginal column totals	12	28	40 (grand total)

*Table information is provided as follows: observed cell totals (expected cell totals) [chi-square statistic for each cell]*.

There was no difference detected between IG and CG concerning the frequency of opening the reminder messages (*p*-value = 0.59). Values 1–3 indicated “not opening much,” and 4–5 indicated “opened a lot.”

It was found that all the participants who wanted to change their behavior related to caffeine and had a goal/reminder set were more likely to change their behavior successfully after 1-month follow-up (*p* = 0.013), compared to the group who wanted to change but did not have reminders set (Table 10).

**Table 10:** Sub-aim 3: does using goals and reminders help people using eHealth services to change their behavior in the short term (1-month follow-up)? Association found with the *p*-value = 0.013.

	**Reminders set**	**Reminders not set**	**Marginal row totals**
Changed behavior	29 (24.68) [0.76]	5 (9.32) [2]	34
Did not change	16 (20.32) [0.92]	12 (7.68) [2.43]	28
Marginal column totals	45	17	62 (grand total)

*Table information is provided as follows: observed cell totals (expected cell totals) [chi-square statistic for each cell]*.

## Discussion

It is essential to start with the fact that the empirical research in the current study does not measure caffeine consumption or people's behavior to drink coffee; instead it explores their actions based on personalized nutrition information and whether creating goals and reminders, with the help of their digital devices, increases behavioral health toward positive actions in the short term. While the previous studies have shown contrasting results, some claiming that learning about genetic traits would benefit the person's health behavior (14, 17, 25, 28, 33), while others not finding evidence of said benefit (10, 17, 23, 24, 26, 27, 29–32, 34–36).

The empirical part of this study did not find that DTCGT will provide an extra benefit for such a personalized medicine eHealth service, which could be due to the limited sample size in the genetic testing group. In general, people's beliefs were strongly related to the genetic test and behavior change, where 71.8% of all respondents thought that the genetic test helped or would have helped them toward better behavior change. However, previous studies have found that the evidence of genetic testing in behavior change is questionable; there are other factors that could lead to behavior change: capability, opportunity, and motivation (20). The authors of this pilot study find that among all empirical study participants (*n* = 160), the base needs for motivation change were present. The people were genuinely interested in caffeine because they responded to the call in social media, where the main keywords were “genetic testing,” “personalized medicine,” and “caffeine.” The participants also had the capability and opportunity present; since they were using their own digital devices, the tools and software were present already or were offered for free (mobile app MediKeep). The “act” of setting caffeine consumption goals and reminders and sticking to their plan was a relatively easy task when compared to the more substantial diet adjustments or regular visits to facilities (gym or clinic). As suggested by “The Behavior Change Wheel,” the COMB-B system (20), the behavior change can be affected by increasing training, education, and enablement. The current study also affected those areas in participants by offering a piece of potential new information in personalized medicine—a detailed article about caffeine (in Estonian), including pharmacogenetic information and general health benefits or risks. The article was specially tailored for the general audience while maintaining the scientific value: list of references, terms explained, and primary focus on genetic results. While 98.3% of the participants understood the article content, only 69.2% confirmed the information to be new to them.

The relatively high percentage of participants feeling uneasy about reading the article (16.9% of *n* = 172 total with *n* = 27 participants without genetic test results) might be related to the fact that the article created a bit of confusion as it was expected to be read with caffeine genetic test results and genotype. Creating confusion was intentional and expected (because of the hope for educational factors). However, the number of participants who skipped the goal setting and decision making was not measured because it was hard to distinguish between people who thought they needed to change their behavior and participants who did not dare to change their behavior as they did not have their genetic test results. There was no association between IG and CT, i.e., those who set the goals and those who did not.

Most of the participants in the study were women (79.9%). In both study groups, participants with genetic testing (63% women) and people without genetic testing (84.7% women), there were more women, but there was a significantly higher men-to-women ratio in the CG.

Participants who opted in for goals and reminders 36% (*n* = 62/172) were more likely to stick to their goals after 1-month follow-up (*p* = 0.013). The sufficient amount of data confirms the findings of previous studies where eHealth behavior solutions seem to work very well (1–5). There was no statistically significant association found on whether participants with the genetic test would be more likely to opt in for goals and reminders (*p* = 0.79) or would more likely open the reminder messages on their digital devices. However, there is a small indication that DTCGT participants are more likely also to increase their nutrition (caffeine) consumption if they find it beneficial (*p* = 0.046). The general trend for caffeine reduction in the CG might be indicated from the fact that without a genetic test (a personalized decision support), it is safer to reduce caffeine intake rather than increase it—to live healthier and reduce possible risks related to caffeine consumption.

While a 1-month follow-up is considered to be short for behavior change measurement, the authors also found that 63% of people with genetic test results had found out their caffeine metabolism more than 5 months ago. As it was not a measurable factor for this study, it is essential to mention that, while this behavior change eHealth study seemed to work for the short term, it could also work for the long term. As commented by one of the IG participants who changed their behavior in five or more months,

*I can not drink coffee, and genetic test gave me an answer why. The same thing is with strong tea. I can drink liters of Coca, Pepsi, or Red Bull. I quit drinking coffee after my results 5 or more months ago, and I stuck to that before the reminders were offered*.

Once people have adjusted their habits with or without the help of genetic or eHealth service, they might adapt it to their routine without the need for goals and reminders later on. Further studies on the topic for long-term behavior change are needed.

Association was found between those feeling better mentally or physically after 1-month caffeine adjustment in IG (*p* = 0.024) and people who did not feel better or did not know how to answer in IG or CG. While they actually might feel better, they also might feel better based on their self-reported results, because of the self-assurance or self-confirmation of justification to their DTCGT, since it is usually an expensive spending.

In Nielsen et al. (37), 52% confirmed of having heard nothing about DTCGT in Toronto; however, the situation has probably improved over time, and the current status in Estonia is 19.6% of participants know nothing about DTCGT. However, Estonians have been well-educated about genetic testing possibilities recently, as in last year, the national biobank had a major campaign to “gift Estonia with 100 000 new biobank participants to its 100th birthday.” There is a small indicator that some of the empirical study participants confused the DTCGT with their previous biobank donation.

The knowledge about the term “nutrigenomics” had quite similar results in 2019 in Estonia when compared with the study in Toronto in 2012−25.7 and 30% respectively had not heard about the term. Similarly, only 5% had heard about it a lot previously. Similar results were also found for the other questions. However, Estonians had a little bit less expected anxiety about learning their genetic traits.

Suggestions for similar studies would include the tracking of all emails when possible (even when sent individually) to be sure that the participants read or opened them in the first place. The current study does not include why some participants with their DNA raw data did not respond to the second email, while participation in the genetic group was high in general. The following questions remain: whether participants felt insecure, whether participation was going to be difficult due to the sharing/signing/uploading request, or whether they did not receive the email.

As the central hypothesis of this study remains unanswered, further research is needed on larger study samples. The authors of this study suggest doubling the number of participants for more accurate statistical analysis: starting from 214 to at least 400.

### Study Limitations

The genotype and caffeine metabolism information for the IG in this empirical research may not have been new for the target group. Many DTCGT companies offer caffeine metabolism information in nutrition reports; it may be a reason why some participants in the IG chose the option not to change anything in their behavior because they already did so a while ago and it has been a routine ever since. On the other hand, the CG got an article focusing on genetic information, and it might have been the reason for the participants without a genetic test to not set a personal goal.

The study group was relatively small, starting with *n* = 213 participants along with *n* = 46 with DTCGT raw data and finishing with *n* = 160 responses. The 1-month follow-up is considered to be short, and no long-term results were measured; however, over half of the participants with genetic test results got their first caffeine-related results more than 5 months ago from the third questionnaire. Most of the answers were self-reported by the participants, while only technical data were obtained elsewhere.

The study faced several technical problems: at some point, some of the reminders or invitations to questionnaires sent by email ended up in the spam filter. Secondly, there was a technical problem in sending out Android reminders in the first week; however, it did not seem to interrupt the results as they did receive messages at least 10 times in 1 month afterward.

As several aims or hypotheses have been investigated and several tests have been run, the resulting statistical data should be considered with some caution. Bonferroni correction should be considered to correct for multiple testing.

### Study Strengths

The current study is the first known study of its kind in Estonia, and probably globally, including DTCGT, behavior change, and eHealth applications. The participants were not aware of the study aims. However, they were genuinely interested in using eHealth interventions.

## Conclusions

As genetic testing has become more affordable for the general public and has been accessible for everyone who wants to get tested via DTCGT companies, the following question remains: whether in the rise of eHealth apps and behavior change programs, the personalized genetic trait disclosure would add an extra benefit for the person's health behavior. While some studies prove it actionable, others remain uncertain. While the authors of this study dug deeper into the question, it was revealed based on the previous meta-analysis of 18 studies worldwide that participating in the study or eHealth program shows positive results in health behavior.

The most active association found in the empirical part of this study was related to the idea claiming that the eHealth applications work (1–5) for behavior change in the short term. However, it was not possible to find differentiation between the DTCGT group and the CG, in adding up for the behavior change, because of the insufficient amount of data. So, the following question remains: whether genetic testing for behavior health change is beneficial. The IG also represented more male participants aged 30–39.

The secondary findings created additional conclusions where people with genetic test results were more likely to increase caffeine intake if they found caffeine to be beneficial or not harmful. They were feeling better (mentally, physically, or both) after their decision to change their behavior related to caffeine in 1 month. So in light of the questions of whether one should increase the nutrition intake or whether they need to decide on a specific nutrition to feel better, genetic testing could be considered. Further studies among larger study groups are needed to have a better understanding of behavior health change in personalized medicine. eHealth applications for short-term behavior change have a positive effect, regardless of a previous genetic disclosure.

## Data Availability Statement

The raw data supporting the conclusions of this article will be made available by the authors, without undue reservation.

## Ethics Statement

The studies involving human participants were reviewed and approved by The study has Research Ethics Committee of the University of Tartu approval no: 290/T-10 since 8.03.2019 until 30.06.2019. The patients/participants provided their written informed consent to participate in this study.

## Author Contributions

KA: main work and research, empirical study. MV: supervision and reviews. AK: forming the study aim questions and mentoring for the statistical calculations. All authors contributed to the article and approved the submitted version.

## Conflict of Interest

The authors declare that the research was conducted in the absence of any commercial or financial relationships that could be construed as a potential conflict of interest.

## Footnotes

^1^MediKeep (https://www.medikeep.eu) is a mobile app for home pharmacy management, and during the study, the company offered a research platform based on active ingredients in caffeine. The functionality included creating an account and receiving reminders within the app and mobile and email notifications.

^2^https://www.theverge.com/2016/3/15/11241334/genetic-testing-disease-risk-dna-behavior-changes.

^3^ https://massive.io

^4^ https://www.id.ee

^5^ https://www.typeform.com

^6^ https://www.socscistatistics.com/tests

## Abbreviations

AI: Artificial Intelligence
DNA: Deoxyribonucleic Acid
DTC: Direct to Consumer
DTCGT: Direct-to-Consumer Genetic Testing
FTO: Fat Mass and Obesity Associated
REVEAL: The Risk Evaluation and Education for Alzheimer's Disease
SNP: Single-Nucleotide Polymorphisms.
