# Supplementary material for: Disclosing Pharmacogenetic Feedback of Caffeine via eHealth Channels, Assessment of the Methods and Effects to Behavior Change: A Pilot Study
Source: Front Digit Health. 2021 Feb 9;2:567656. doi: 10.3389/fdgth.2020.567656 (PMC8521856; doi:10.3389/fdgth.2020.567656)
Supplement: Supplementary file 1 [file Data_Sheet_1.PDF]

Study Design

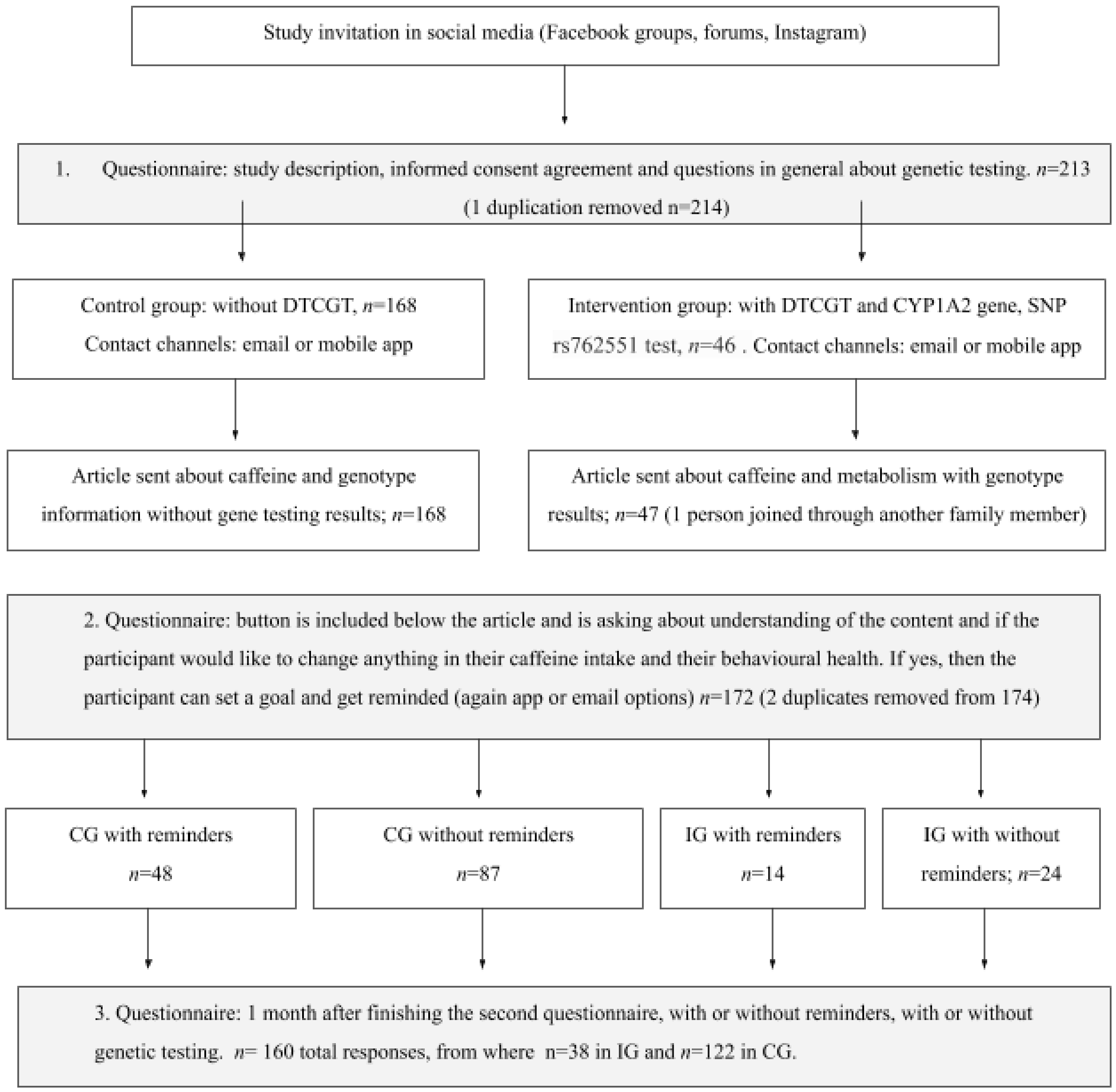

Invitation to the study and I-III questionnaires

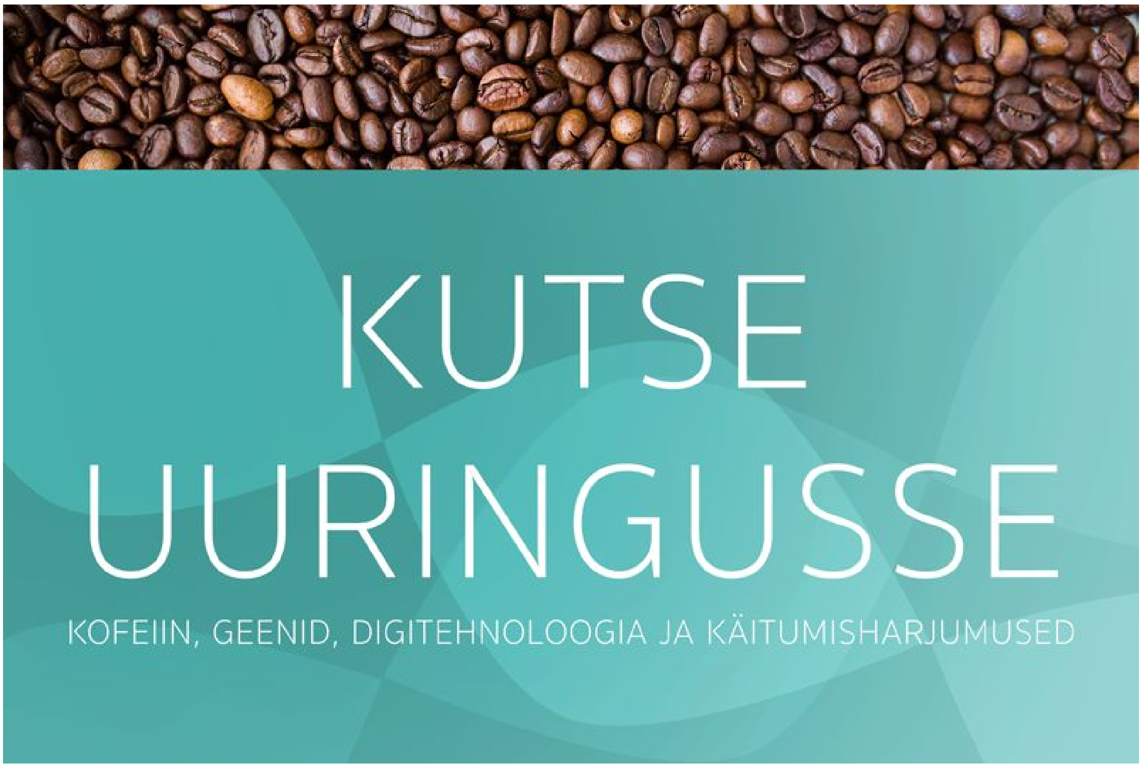

Figure 9. Banner used in social media along with the invitation text.

Invitation in social media (translated from estonian):
"I invite you to my Master's thesis study! The study compares the effectiveness of the methods used to deliver digitally acquired health and gene information. You are suitable for the study if you are at least 18 years of age and are interested in topics such as genetic tests, caffeine (found in coffee and, for example, green tea) and personalized medicine. Come and read more on the research page."

Invitation and description in the beginning of online questionnaire:

"Hello, my name is Kerti Alev, and I do my Master's thesis at the Tallinn University of Technology in the field of Health Care Technologies. I invite you to a study on health behavior, genes, and digital technology. The topic of my Master's thesis is: "Genetic Feedback through Digital Channels in Personalized Medicine, Methods and Changes in Behavior." Future medicine is increasingly engaging in digital decision support channels that allow for more personal health problem solving or prevention. Genetics will play an

ever-increasing role, especially if it is linked to human health indicators. For my research, I have chosen the active ingredient caffeine as it is widely used and its genetical metabolism is easily identifiable. You can get information by email or through the MediKeep mobile app. The user experience I suggest you could apply in the future to many other nutrients, medicines, or preventable diseases. The study runs from March to June 2019. You are suitable for the study if you are at least 18 years old and answer YES to at least one of the following question:

1. Consume caffeine regularly (coffee, green tea, cocoa, and certain soft drinks).
2. You have done a genetic test and/or have your own DNA raw data file (which you are willing to share with me).
3. You haven't done a genetic test.
4. You do not consume caffeine daily (but you are interested in the subject).

A maximum of three questionnaires should be completed during the study (two at the beginning and the third at the end of the study) and one article about caffeine, and its genetic suitability should be read. If you have been taken a genetic test before and you have shared your data with me, the result of the gene test will be shown next to the article. After reading the article, you should decide whether or not to change your caffeine intake. You can then set up a goal and reminder for changing behavior, but it is not mandatory. If you want a reminder, you can choose between an email or a MediKeep mobile app (the app is available for free on iOS and Android). Reminders are sent up to five times a week and within one month, and you can design your own goal and message content (more or less caffeine).

If you wish, you can remain anonymous in the study, but you should use an anonymous email to communicate. Questions relate to your blood pressure, your friendship with caffeine (coffee, green tea, etc.) and genetic tests (I can also ask for a DNA raw data file if you are a 23and, Ancestry, MyHeritage, MediKeep client). Your DNA raw data transfer is encrypted (via DigiDoc, the instructions and the information needed for encryption is forwarded via email) and data over the Internet (kerti.portal.massive.app). I keep your data encrypted and secure on my private computer, which is password protected and in a folder that is in turn password protected, where no one else has access. Confidentiality is guaranteed to all subjects. The data is stored until September 2019. Participation is strictly voluntary and does not offer any tangible benefits. Study subjects do not benefit directly from participation in

research, but the topic and field could be of interest to the participants and provide new knowledge about genetics, health and the relationship between active ingredients.

Risks: Participation in the study should not cause you any harm to your health because the consumption of caffeine is part of our daily routine, but problems with caffeine consumption may occur. Although studies have shown that humans have positive features of 250 mg caffeine doses, such as awakening, increased attention, focus and well-being; then the dose of 500 mg may increase nervousness, anxiety, agitation, irritability, nausea, tremors, sweating, palpitations, restlessness and perhaps dizziness. Amount from ~7 to 10 mg/kg may cause symptoms in the average adult such as chills, flushing, nausea, headache, palpitations, and tremors. Individual reactions may vary. If you experience any of these side effects during the study period, caffeine intake should be significantly reduced or stopped. You may indicate during the study period that you wish to opt out of the study and that the data related to you will be deleted and/or not used. The survey results and conclusions can be used to develop the services of MediKeep OÜ.

The study is authorized by the University of Tartu Ethics Committee of Human Studies, no: 290 / T-10 from 05.03.2019 to 30.06.2019. ”

Before asking questions and after clicking on “Start”: “I have been informed about the above mentioned study and I am aware of the purpose of the research and the research methodology and risks. I confirm my agreement to participate in it and to process your data. If I have my own DNA raw data file, I will give permission to use my data in the survey.” (Mandatory check box).

Questionnaire I

1. Name
2. Email
3. Age: 30-39; 40-49; 18-29; 50-59; 60-69; 70-70 and more
4. Ethnicity: European origin; african, asian, other
5. Education level: primary education, secondary education, Bachelor, Master, Doctor
6. How much have you heard of commercially available genetic testing (advertising, social media, friends, co-workers, etc.): nothing, I've heard something, I have heard a lot about private company genetic tests

7. How much have you heard of nutrigenomics and nutrigenetics (a science that studies the links between genes, health, and nutrition): nothing , a little bit, I've heard something, I've heard a lot
8. I'm interested how genes affect diet: I fully agree, I agree to some extent, neither agree or disagree, rather disagree, disagree strongly
9. I would benefit from learning how my genes affect my diet: I fully agree, I agree to some extent, neither agree or disagree, rather disagree
10. When I get (got) to know my genetic profile, it affects (has influenced) my eating habits: I fully agree, I agree to some extent, neither agree or disagree, rather disagree
11. Getting to know the genetic profile causes (caused) discomfort / anxiety to me: I fully agree, I agree to some extent, neither agree or disagree, rather disagree
12. I'd do a genetic test (or I've done) to learn more about myself: I fully agree, I agree to some extent, neither agree or disagree, rather disagree
13. I'd do (I've done) a genetic test to better understand what I should change to be healthier: I fully agree, I agree to some extent, neither agree or disagree, rather disagree
14. I'd do (I did) a gene test so the doctor could give me better suggestions: I fully agree, I agree to some extent, neither agree or disagree, rather disagree
15. How do you prefer I contact you for the next experiment: email, mobile app MediKeep, both
16. *Name*, do you have your DNA raw data: yes, no, I am not sure
17. Which service provider have you performed a genetic test: 23andme.com, Anchestry.com, MyHeritage.com, MediKeep.eu, MyLivingDNA.com, Geni.com
18. Thanks, ! I'll contact you soon! If you find out how to share the study, I would be very grateful!

Questionnaire II (after reading the article about caffeine genetic metabolism)

1. Email
2. After reading the article, I felt insecure: yes, no. I don’t know
3. I understood what the caffeine article explained to me: yes, no
4. The article's suggestions were news to me that I didn't know before: yes, no
5. I liked the results of my article/report: yes, no. I don’t know
6. The article/report results will help me make better choices: yes, no. I don’t know
7. I wish to know more: yes, no

8. Would you like to change something about your health behavior? Do we set a goal and a reminder for you? (caffeine related): yes, no
9. I want to add a weekly reminder (maximum 5x per week in 1 month). Description: The frequency of messages is selected by Kerti depending on the answers to the next question. But reminders come rather less than more frequently): I want to consume less food containing caffeine; I want to consume more caffeine-containing food
10. Choose your product: Coffee, Tea, Cocoa, Chocolate, Other
11. In order to make your reminder more personal, write down what you want the caffeine-containing food to consume more or less (some of your favorite products), why (related to some health condition) and when would you like to receive it (after lunch, morning or evening)? You can also set multiple goals. Description: example "Set your goal to drink more coffee than usual - if you feel like it you should take a cup!" Or "Set your goal to drink less coffee than usual - skip your next cup of coffee now!" (open ended question)
12. I want personalized reminders via these channels: e-mail, mobile app MediKeep (available for free on iOS and Android smartphones, login or create an account with the same email you used here).
13. Mobile: Andoid, iOS
14. Option end A: Thank you! To receive reminder via phone please download MediKeep free app for Android, create account or log in and check whether "notifications" are enabled.
15. Option end B: Thank you! The third questionnaire will come to you in one month.

Questionnaire III (follow-up in one month)

1. Email
2. When Did You Know Your Caffeine Metabolism Results? Description: If you already received CYP1A2 results, choose the appropriate answer. If you got an article without a genetic test, please choose the appropriate answer: I did not participate with the genetic test, up to 2 months ago, 3-4 months ago, 5 months or more.
3. Did you wanted to change your behavior after reading the article: Yes, I wanted to change my caffeine consumption habits compared to the past; No, I'm not in contact with caffeine, so I should change something; No. I thought I was already consuming caffeine in appropriate amounts for myself; I couldn't change anything based on what I read.

4. Do you feel better after adjusting your behavior to caffeine: mentally; physically; mentally and physically; I don't know; no I do not
5. Did you set a reminder / goal in the study (email, MediKeep app): yes, no, I do not remember
6. Technical Control Question: Reminders reached your email and / or MediKeep mobile app: yes, no
7. Did you notice the emails that came to your email or to the MediKeep mobile app: yes, no
8. How long did the reminders follow? 0 = not watched at all; 4 = I was watching everyone until the end. Reminders had to be received during the one month.
9. Have you opened reminders (email or app opened after notification)? 0 = never opened; 4 = I opened every time.
10. Do you think that reminders are or would have been useful to change your behavior: yes, no, I don't know.
11. Do you think that the gene test had (would have) benefited to change your behavior: yes, no, I don't know.
12. Did you change your behavior with caffeine within one month after reading the article?

    I changed my habits and I've followed my decision to this day.

    I changed in the beginning, but I'm back with my old habits today.

    I wanted to change, but it didn't work.

    Didn't want and didn't change.
13. Would you like to experience this exchange of information / user experience on other genetic traits (other nutrients, disease prevention, drug suitability): yes, no, I don't know
14. Do you want to leave a comment on participation in the survey? (open ended)
15. Thank you!
